# A ferroptosis–based panel of prognostic biomarkers for Amyotrophic Lateral Sclerosis

**DOI:** 10.1038/s41598-019-39739-5

**Published:** 2019-02-27

**Authors:** David Devos, Caroline Moreau, Maeva Kyheng, Guillaume Garçon, Anne Sophie Rolland, Hélène Blasco, Patrick Gelé, T. Timothée Lenglet, C. Veyrat-Durebex, Philippe Corcia, Mary Dutheil, Peter Bede, Andreas Jeromin, Patrick Oeckl, Markus Otto, Vincent Meninger, Véronique Danel-Brunaud, Jean-christophe Devedjian, James A. Duce, Pierre François Pradat

**Affiliations:** 1 Department of Neurology, ALS Center, Lille University, INSERM UMRS_1171, University Hospital Center, LICEND COEN Center, Lille, France; 2 Department of Medical Pharmacology, Lille University, INSERM UMRS_1171, University Hospital Center, LICEND COEN Center, Lille, France; 3 Department of Biostatistics, Lille University, University Hospital Center, Lille, France; 4 Univ. Lille, CHU Lille, Institut Pasteur de Lille, EA4483 IMPECS-IMPact de l’Environnement Chimique sur la Santé humaine, Lille, France; 5 Université François-Rabelais, Inserm U930, Laboratoire de Biochimie, CHRU de Tours, France; 6 CRB/CIC1403, Université de LILLE, Lille, France; 7 APHP, Department of Neurophysiology, Pitié-Salpêtrière Hospital, Paris, France; 8 Centre Constitutif SLA, Tours-Fédération des centres SLA Tours-Limoges, LITORALS, Tours, France; 9 Biomedical Imaging Laboratory, CNRS, INSERM, Sorbonne University, Paris, France; 10 Computational Neuroimaging Group, Academic Unit of Neurology, Trinity College Dublin, Dublin, Ireland; 11 Quanterix, Lexington, Massachusetts, USA; 12 Department of Neurology, Ulm University Hospital, Oberer Eselsberg 45, 89081 Ulm, Ulm, Germany; 13 APHP, Department of Neurology, Paris ALS Center, Pitié Salpêtrière Hospital, Paris, France; 14 ALBORADA Drug Discovery Institute, University of Cambridge, Cambridge Biomedical Campus, Hills Road, Cambridge, CB2 0AH UK; 15 School of Biomedical Sciences, Faculty of Biological Sciences, University of Leeds, Leeds, West Yorkshire United Kingdom; 16 Northern Ireland Centre for Stratified Medicine, Biomedical Sciences Research Institute Ulster University, C-TRIC, Altnagelvin Hospital, Derry/Londonderry, United Kingdom

## Abstract

Accurate patient stratification into prognostic categories and targeting Amyotrophic Lateral Sclerosis (ALS)-associated pathways may pave the way for promising trials. We evaluated blood-based prognostic indicators using an array of pathological markers. Plasma samples were collected as part of a large, phase III clinical trial (Mitotarget/TRO19622) at months 1, 6, 12 and 18. The ALSFRS-r score was used as a proxy of disease progression to assess the predictive value of candidate biological indicators. First, established clinical predictors were evaluated in all 512 patients. Subsequently, pathologic markers, such as proxies of neuronal integrity (Neurofilament light chain and phosphorylated heavy chain), DNA oxidation (8-oxo-2′-desoxyguanosine), lipid peroxidation (4-hydroxy-2-nonenal, isoprostane), inflammation (interleukin-6) and iron status (ferritin, hepcidin, transferrin) were assessed in a subset of 109 patients that represented the whole cohort. Markers of neuronal integrity, DNA and lipid oxidation, as well as iron status at baseline are accurate predictors of disability at 18-month follow-up. The composite scores of these markers in association with established clinical predictors enable the accurate forecasting of functional decline. The identified four biomarkers are all closely associated with ‘ferroptosis’, a recently discovered form of programmed cell death with promising therapeutic targets. The predictive potential of these pathophysiology-based indicators may offer superior patient stratification for future trials, individualised patient care and resource allocation.

## Introduction

Amyotrophic lateral sclerosis (ALS) is a relentlessly progressive neurodegenerative condition with no effective disease-modifying therapy. Late recruitment to pharmacological trials, clinical heterogeneity, and lack of specific monitoring markers are some of the main barriers to successful drug development. Accurate patient stratification into prognostic categories^1^ and targeting ALS-associated pathways may pave the way for promising phase II trials. Reliance on easily accessible biofluids and the appraisal of markers that are directly implicated in ALS pathogenesis is a key strategy for effective biomarker development. Ferroptosis^2^ in motor neurons is increasingly recognised as an important process of ALS^3^ with lipid and iron accumulation being surrogate markers for this type of programmed cell death. Neurofilament light chain (NfL) and phosphorylated heavy chain (pNfH) are well established markers of neural integrity in ALS^4–9^. Oxidised DNA products (oxidation (8-oxo-2′-desoxyguanosine (8-oxo-dG))^4,10,11^, and lipids (4-hydroxy-2-nonenal; 4-HNE and isoprostane)^10,12^ have also been shown to be consistently elevated in ALS. Lastly, interleukin-6 (IL-6)^13,14^ as well as ferritin (FT)^4,15–19^, hepcidin and transferrin are accepted markers of inflammation and iron metabolism respectively.

The biomarkers were assessed in the Mitotarget/TRO19622 study, a cohort of 512 ALS patients from 15 European centers partaking in a negative, randomized, double-blinded, placebo-controlled phase III trial of olesoxime (NCT:00868166)^20^. First we analysed the demographic, clinical and biological safety parameters on disease progression (i.e. functional assessment (ALSFRS-r)) for the whole cohort. Then, to enable longitudinal functional assessment we assessed a ferroptosis–based panel of prognostic biomarkers in a subgroup of 109 patients that was randomly selected from the 286 patients who had completed the 18-month-follow up assessment. We focused on baseline parameters, which are convenient to establish patient stratification into prognostic categories. The recently identified candidate predictors^1^ were modelled to identify a new panel of prognostic indicators and contrast them against clinical predictors typically used as a gold standard.

## Results

The baseline clinical characteristics of the two study populations, that culminated in an entire trial cohort of 512 patients and a subset of 109 patients, were comparable (Table e-1**)**. No effect of olesoxime was observed on any of the parameters. Safety parameters were not associated with disease progression in the entire trial cohort of 512 patients or in the subset of 109 patients. Only creatine phosphokinase was associated with ALSFRS-r score at a given time-point, without a prognosis value (Tables e-2 and e-3).

**Figure 1:**
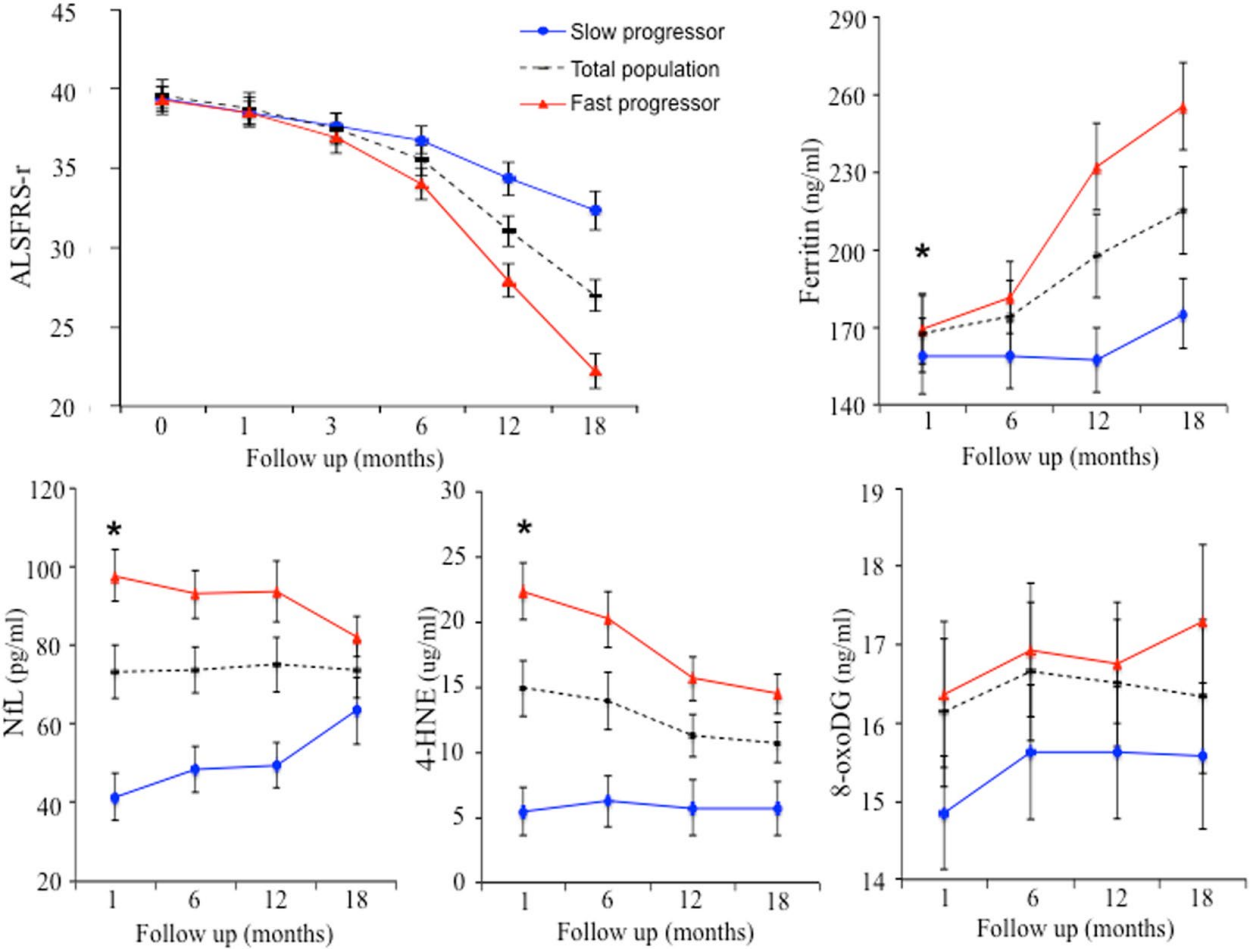
Progression of the specific biomarkers over 18 months in fast versus slow progressors. The association of specific parameters at baseline with ALSFRS-r progression was analyzed by considering two groups of disease decline; slow and fast. The population was divided according to a median in the ALSFRS-r score decrease rate from time of inclusion to 18 months. The distribution of each parameter (means and SEM) over time was compared between the two groups of slow (54 patients) and fast progressors (n = 55 patients) using Mann-Whitney U tests. *p-value < 0.05.

The ALSFRS-r scores showed a mean reduction of 0.70 point per month over the 18-month period. NfL, pNfH, 4-HNE, 8-oxo-dG and FT at baseline were negatively associated with ALSFRS-r at follow-up (Table 1), i.e. higher baseline values indicate a more significant functional disability at 18-month follow-up. Hepcidin, Transferrin, IL-6 and isoprostane were not significantly associated (Table 1). Similar results were found after adjusting for baseline characteristics (main clinical and biological data). In multivariate analyses, baseline NfL, 4-HNE, 8-oxo-dG and FT were independently associated with ALSFRS-r decline (Table 2 with the equation of prediction). From a subset of patients we next assessed these biomarkers in two groups of disease decline. In accordance to a median in the ALSFRS-r score decrease rate from time of inclusion to 18 months, these were referred to as ‘slow’ or ‘fast’ progressors. The ‘fast- progressors’ (n = 55 patients, mean monthly reduction of 0.94 point at ALSFRS-r score) had significantly higher values of NfL, 4-HNE and FT compared to ‘slow-progressors’ (54 patients, mean monthly reduction of 0.33 point at ALSFRS-r score) at baseline. These differences in NfL and 4-HNE progressively decreased with disease progression (Fig. 1). Conversely, the difference of FT became higher with a greater variability as disease progressed. No significant difference was observed with 8-oxo-dG levels.

**Table 1:** Specific baseline parameters on ALSFRS-r progression.

Factors at baseline	Unadjusted		Adjusted*	
	Coefficient β ± SE	P-value	Coefficient β ± SE	P-value
NfL^a^	−0.05 (0.005)	<0.001	−0.03 (0.005)	<0.001
pNfH^a^	−0.01 (0.001)	<0.001	−0.006 (0.001)	<0.001
4-HNE^a^	−0.16 (0.01)	<0.001	−0.15 (0.01)	<0.001
8-OHdG	−0.02 (0.004)	<0.001	−0.02 (0.003)	<0.001
Ferritin^a^	−0.006 (0.002)	0.005	−0.006 (0.002)	0.001
Hepcidin^c^	−0.02 (0.01)	0.083	−0.01 (0.01)	0.27
Transferrin^b^	−0.0001 (0.003)	0.97	−0.0001 (0.003)	0.96
IL-6	0.001 (0.002)	0.65	−0.002 (0.002)	0.46
Isoprostane	0.12 (0.07)	0.073	0.07 (0.06)	0.26

Specific parameters were evaluated on an allocated treatment group. Linear mixed models with random intercept before and after adjustment to baseline characteristics associate with ALSFRS-r progression (p < 0.10 for their interaction with time in multivariate analysis). *Adjusted on treatment and pre-specified baseline factors with their interactions to time (BMI, MMT, SVC, sodium and time since the onset of clinical signs). ^a–c^A coefficient corresponding to the effects of a respective 10, 1000 and 10000 point increase.

**Table 2:** A final model of specific baseline parameters associated with ALSFRS-r progression.

Factors at baseline	Effect on ALSFRS-r progression	
	Coefficient β ± SE	p
NfL^a^	−0.02 (0.005)	0.004
4-HNE^a^	−0.11 (0.02)	<0.001
8-OHdG	−0.01 (0.004)	<0.001
Ferritin^a^	−0.01 (0.002)	<0.001

All parameters associated with ALSFRS-r progression from the adjusted models shown in Table 1 (interaction with time < 0.10) were included in a multivariable linear mixed model. Neurological parameters were removed manually using the same backward selection approach. The multivariate analysis was performed on the population for specific parameters using the final mixed model. ^a^a coefficient corresponding to the effects of a 10 point increase. Analysis was adjusted for treatment and pre-specified baseline factors with their interactions to time (BMI, MMT, SVC, sodium and time since the onset of signs).

Examples of prediction of the monthly rate of reduction of ALSFRS-r:

SVC (70%), diagnosis delay (12 months), BMI (24), MMT (140) *(sodium: 140)*:

- NfL (100) + 4-HNE (20) + 8-oxo- dG (17) + Ferritin (170) = monthly adjusted rate: −0.72.

- NfL (70) + 4-HNE (15) + 8-oxo- dG (16) + Ferritin (160) = monthly adjusted rate: −0.65.

- NfL (40) + 4-HNE (5) + 8-oxo- dG (14) + Ferritin (150) = monthly adjusted rate: −0.56.

At 18 months the cohort had a mean reduction per month of 2 points in MMT, 1.5 points in SVC and 0.06 points in BMI. NfL and 4-HNE had a negative association with MMT (p < 0.001 and p = 0.021 respectively) i.e. higher values at baseline indicated lower MMT at follow-up. NfL also negatively correlated with SVC (p = 0.010). Conversely, baseline FT had a positive association with SVC (p = 0.039) and BMI (p = 0.002) at follow-up. No association was identified between disability at follow-up and the inflammatory marker IL-6 at baseline.

## Discussion

In comparison to established clinical predictors, this longitudinal study demonstrates the predictive value on disease progression using four easy quantifiable blood biomarkers. Higher NfL, 4-HNE, 8-oxo-dG and FT levels at baseline were associated with greater ALSFRS-r decline over the 18-month follow-up period. Interestingly, the changes of these parameters over time preceded functional decline (i.e. difference between ‘fast’ and ‘slow’ progressors occurred at 6 months, Fig. 1). The persistently elevated values of these markers in the fast-progressor population suggest relentless neuronal degeneration during the 18-month follow-up. Given the possible predictive value of these biomarkers, they may aid patient stratification for future phase trials. From a clinical perspective, they may also contribute to precision care planning, resource allocation and management of individual patients.

The nervous system is particularly rich in lipids and products of lipid peroxidation such as 4-HNE may represent an important and currently under evaluated proxy of disease activity. It is noteworthy that the highly reactive cytotoxic 4-HNE irreversibly cross-links proteins such as neurofilaments^21^. Changes in FT, an indicator of brain iron status, may represent an additional aetiological factor promoting free radical production. Increased lipid peroxidation and iron accumulation are key components of iron dependent programmed cell death; ferroptosis^2^.

In conclusion, our findings indicate that markers of ferroptosis in ALS are associated with clinical decline. Elevated NfL and 8-oxo-dG levels on the other hand are secondary to axonal skeleton disintegration and DNA fragmentation, likely a downstream effect of ferroptosis. These observations need to be replicated in larger populations and the predictive value of these markers need to be examined on survival. The characterisation of these mechanisms and the development of ferroptosis-based markers is particularly timely, as iron chelation^4^ and anti-ferroptotic therapy^2,22^ are currently under investigation for a range of neurodegenerative conditions including ALS.

## Methods

Mitotarget/TRO19622 was a negative, randomized, double-blinded, placebo-controlled phase III trial for olesoxime (NCT:00868166) that included 512 ALS patients from 15 European centers^20^. All experiments were performed in accordance with French and European guidelines and regulations. Following approval from a local ethics committee at Assistance Publique Hôpital Pitié-Salpêtrière and informed consent from each participant, data were collected every 3 months during the 18-month trial period. Participants were diagnosed with either ‘probable’ or ‘definite’ ALS according to the revised El Escorial criteria, and only patients with symptom duration of more than 6 and less than 36 months were enrolled. In addition to riluzole, patients received olesoxime or placebo.

A subgroup of 109 patients was randomly selected from the 286 patients that completed the 18-month-follow up assessment. This enabled longitudinal functional assessment (ALSFRS-r)^23^, but precluded survival analyses. All recently identified candidate predictors^1^ (Table e-1) were included in a prediction model with the exception of frontotemporal dementia (due to a lack of phenotype in this cohort) and the presence of *C9orf72* hexanucleotide repeat expansions (data not available).

Finally the population was also divided into two groups of disease decline (i.e. slow and fast), according to a median in the ALSFRS-r score decrease rate from time of inclusion to 18 months.

Plasma samples were obtained at 1, 6, 12 and 18 months after enrolment. Standard ‘Safety parameters’ were monitored during the trial (Table e-2). The ‘Specific parameters’ were measured in duplicate using commercially available kits for NfL (NF-light Kit Advantage, Quanterix, Lexington, MA, USA), pNfH (Neurofilament ELISA, Euroimmun AG, Lübeck, Germany), 8-oxo-dG (ELISA Kit, Abcam, Cambridge, UK: ab201734), 4-HNE (OxiSelect™ HNE Adduct Competitive ELISA Kit, Cell Biolabs, Inc., San Diego, CA, USA: STA-838), 8-isoprostane (ELISA Kit, Abcam, Cambridge, UK: ab175819), interleukin-6 (Human Magnetic Luminex Screening Assay, R&D Systems - Bio-Techne, Lille, France:HUVF4Lrv), ferritin (human ELISA Kit, Abcam, Cambridge, UK: ab108698), transferrin (human ELISA Kit, Abcam, Cambridge, UK: ab108911) and hepcidin (Human Quantikine ELISA Kit, R&D Systems - Bio-Techne, Lille, France: DHP250).

## Statistical analyses

The predictive value of the clinical and ‘safety parameters’ on the ALSFRS-r score was investigated using bivariate linear mixed models with randomized coefficients (Table e-2). The fixed effects in the model included time, baseline characteristics and their interaction. All baseline characteristics that associated either alone (p < 0.05) or in interaction with time (p < 0.10) were included in a multivariable linear mixed model (Table e-3**)**.

The predictive value of the ‘specific parameters’ on the ALSFRS-r score was investigated using linear mixed modelling. Random intercept was performed before and after adjustment to the baseline characteristics associated with ALSFRS-r progression and allocated treatment group (Table 1). All parameters associated with ALSFRS-r progression in the adjusted models (interaction with time < 0.10) were included in the multivariable linear mixed model (Table 2).

Finally, the association between specific parameters at baseline and progression of other parameters (e.g MMT, SVC, BMI) were investigated by bivariate linear mixed modelling with random intercept.

All statistical tests were performed at the 2-tailed α level of 0.05. Data were analysed using SAS version 9.4 [SAS Institute Inc., Cary, NC 27513, USA].

## Supplementary information

Supplemental Tables

## Data Availability

All the anonymized data and the statistical analyses will be shared by request from any qualified investigator.
