# Author Correction: A ferroptosis–based panel of prognostic biomarkers for Amyotrophic Lateral Sclerosis

**DOI:** 10.1038/s41598-020-58956-x

**Published:** 2020-02-19

**Authors:** David Devos, Caroline Moreau, Maeva Kyheng, Guillaume Garçon, Anne Sophie Rolland, Hélène Blasco, Patrick Gelé, T. Timothée Lenglet, C. Veyrat-Durebex, Philippe Corcia, Mary Dutheil, Peter Bede, Andreas Jeromin, Patrick Oeckl, Markus Otto, Vincent Meininger, Véronique Danel-Brunaud, Jean-Christophe Devedjian, James A. Duce, Pierre François Pradat

**Affiliations:** 1 Department of Neurology, ALS Center, Lille University, INSERM UMRS_1171, University Hospital Center, LICEND COEN Center, Lille, France; 2 Department of Medical Pharmacology, Lille University, INSERM UMRS_1171, University Hospital Center, LICEND COEN Center, Lille, France; 3 Department of Biostatistics, Lille University, University Hospital Center, Lille, France; 4 Univ. Lille, CHU Lille, Institut Pasteur de Lille, EA4483 IMPECS-IMPact de l’Environnement Chimique sur la Santé humaine, Lille, France; 5 Université François-Rabelais, Inserm U930, Laboratoire de Biochimie, CHRU de Tours, France; 6 CRB/CIC1403, Université de LILLE, Lille, France; 7 APHP, Department of Neurophysiology, Pitié-Salpêtrière Hospital, Paris, France; 8 Centre Constitutif SLA, Tours-Fédération des centres SLA Tours-Limoges, LitORALS, Tours, France; 9 Biomedical Imaging Laboratory, CNRS, INSERM, Sorbonne University, Paris, France; 10 Computational Neuroimaging Group, Academic Unit of Neurology, Trinity College Dublin, Dublin, Ireland; 11 Quanterix, Lexington, Massachusetts USA; 12 Department of Neurology, Ulm University Hospital, Oberer Eselsberg 45, 89081 Ulm, Ulm Germany; 13 APHP, Department of Neurology, Paris ALS Center, Pitié Salpêtrière Hospital, Paris, France; 14 ALBORADA Drug Discovery Institute, University of Cambridge, Cambridge Biomedical Campus, Hills Road, Cambridge, CB2 0AH UK; 15 School of Biomedical Sciences, Faculty of Biological Sciences, University of Leeds, Leeds, West Yorkshire United Kingdom; 16 Northern Ireland Centre for Stratified Medicine, Biomedical Sciences Research Institute Ulster University, C-TRIC, Altnagelvin Hospital, Derry/Londonderry, United Kingdom

Correction to: *Scientific Reports* 10.1038/s41598-019-39739-5, published online 27 February 2019

The original version of this Article contained a typographical error in the spelling of the author Vincent Meininger, which was incorrectly given as Vincent Meninger. This has now been corrected in the PDF and HTML versions of the Article, and in the accompanying Supplementary Information file.

