# Supplementary material for: A ferroptosis–based panel of prognostic biomarkers for Amyotrophic Lateral Sclerosis
Source: Sci Rep. 2019 Feb 27;9:2918. doi: 10.1038/s41598-019-39739-5 (PMC6393674; doi:10.1038/s41598-019-39739-5)
Supplement: Supplementary file 1 — Supplemental Tables [file 41598_2019_39739_MOESM1_ESM.docx]

**A ferroptosis–based panel of prognostic biomarkers for Amyotrophic Lateral Sclerosis**

David Devos MD, PhD ^1,2^ Caroline Moreau, MD,PhD*^1^, Maeva Kyheng*^3^, Guillaume Garçon, PhD ^4^, Anne Sophie Rolland, PhD ^2^, PhD, Hélène Blasco, PharmD, PhD^5^, Patrick Gelé, PhD^6^, Timothée Lenglet T, MD^7^, Veyrat-Durebex C, PhD^5^, Philippe Corcia, MD,PhD^8^, Mary Dutheil^2^, Peter Bede MD, PhD^9,10^Andreas Jeromin PhD^11^, Patrick Oeckl PhD^12^ Markus Otto MD^12^, Vincent Meninger MD^13^ Véronique Danel-Brunaud, MD^1^, Jean-christophe Devedjian, PhD ^2^ James A. Duce, PhD ^14, 15^, Pierre François Pradat, MD,PhD^9,16^

*Authors contributed equally

**1** Department of Neurology, ALS Center, Lille University, INSERM UMRS_1171, University Hospital Center, LICEND COEN Center, Lille, France

**2** Department of Medical Pharmacology, Lille University, INSERM UMRS_1171, University Hospital Center, LICEND COEN Center, Lille, France

**3** Department of Biostatistics, Lille University, University Hospital Center, France

**4** Univ. Lille, CHU Lille, Institut Pasteur de Lille, EA4483 IMPECS-IMPact de l’Environnement Chimique sur la Santé humaine, France

**5** Université François-Rabelais, Inserm U930, Laboratoire de Biochimie, CHRU de Tours, France

**6** CRB/CIC1403, Université de LILLE

**7** APHP, Department of Neurophysiology, Pitié-Salpêtrière Hospital, Paris, France

**8** Centre Constitutif SLA, Tours-Fédération des centres SLA Tours-Limoges, LITORALS

**9** Ramsay, Hôpital des Peupliers, Paris, France

**10** Computational Neuroimaging Group, Academic Unit of Neurology, Trinity College Dublin, Ireland

**11** Quanterix, Lexington, Massachusetts, USA

**12** Department of Neurology, Ulm University Hospital, Oberer Eselsberg 45, 89081 Ulm, Ulm, Germany

**13** APHP, Department of Neurology, Paris ALS Center, Pitié Salpêtrière Hospital, France

**14** ALBORADA Drug Discovery Institute, University of Cambridge, Cambridge Biomedical Campus, Hills Road, Cambridge CB2 0AH, UK.

**15** School of Biomedical Sciences, Faculty of Biological Sciences, University of Leeds, Leeds, West Yorkshire, United Kingdom.

**16** Sorbonne Université, CNRS, INSERM, Laboratoire d’Imagerie Biomédicale, Paris, France; APHP, Département de Neurologie, Hôpital Pitié-Salpêtrière, Centre référent SLA, Paris, France

**Table e-1: Characteristics of patients’ cohorts at baseline**

|  | Population for specific parameters (n=109) | Global cohort for safety parameters (n=512) |
| --- | --- | --- |
| Age of onset | 54.1+/-11.3 | 56.5+/-11.2 |
| Men/women | 69/40 = 1.7 | 331/181 = 1.8 |
| Olexosime/placebo | 55/54 = 1.01 | 259/253 = 1.02 |
| Disease duration (months) | 17.9+/-8.7 | 17.2+/-8.25 |
| Definite/Probable | 22 (20%)/87(80%) | 107 (20%)/404(80%) |
| Diagnosis delay | 10.3+/-6.8 | 10.2+/-5.8 |
| Bulbar/Spinal onset | 24 / 85 = 0.28 | 101 / 411 = 0.24 |
| ALSFRS-r | 39.6+/-4.8 | 38.6+/-5.03 |
| BMI (kg/m^2^) | 24.9+/-3.3 | 24.7+/-3.6 |
| MMT | 131 +/-16.8 | 127 +/-18.4 |
| SVC (%) | 96.1+/-15.6 | 93.1+/-15 |

Mean ± SD [1^st^ quartile- 3^rd^ quartile] are indicated. A Student test or Wilcoxon test was applied on the clinical outcomes according to distribution (normal or not normal). Disease duration represents the months since the patient presented with the first symptoms of the disease, Body mass index (BMI), muscle strength by manual muscle testing (MMT) and Slow Vital Capacity (SVC). The classical safety parameters monitored during a trial are detailed in Table e-2.

**Table e-2: Univariate analysis of demographic, clinical and biological safety parameters on ALSFRS-r and disease progression**

|  | Effect on ALSFRS-r at fixed time or baseline* | | Effect on ALSFRS-r progression | |
| --- | --- | --- | --- | --- |
| Factors at baseline | Coefficient β ± SE | p-value | Coefficient β ± SE | p-value |
| ALSFRS-r progression | -0.96 ± 0.03 | <.0001 | - | - |
| Treatment | 1.02 ± 0.48 | 0.032 | 0.03 ± 0.06 | 0.65 |
| Age at baseline | -0.03 ±0.02 | 0.13 | -0.003 ± 0.003 | 0.22 |
| Gender (Women vs Men) | -2.25 ± 0.49 | <.0001 | -0.07 ± 0.07 | 0.32 |
| Age at onset | -0.02 ± 0.02 | 0.25 | -0.005 ± 0.003 | 0.10 |
| Time since the onset of signs | -0.14 ± 0.03 | <.0001 | 0.03 ± 0.004 | <.0001 |
| Form (Spinal vs Bulbar) | -0.52 ± 0.60 | 0.39 | 0.13 ± 0.08 | 0.11 |
| El Escorial category (Definite vs Probable ALS) | -2.78 ± 0.57 | <.0001 | -0.06 ± 0.08 | 0.44 |
| BMI * | -0.57 ± 0.24 | 0.017 | 0.06 ± 0.03 | 0.045 |
| SVC * | 1.70 ± 0.22 | <.0001 | 0.09 ± 0.03 | 0.002 |
| MMT* | 3.31 ± 0.19 | <.0001 | 0.10 ± 0.03 | 0.003 |
| CPK* | 0.68 ± 0.24 | 0.0003 | 0.04 ± 0.03 | 0.17 |
| Life quality * | 1.43 ± 0.22 | <.0001 | 0.05 ± 0.03 | 0.13 |
| Systolic blood pressure * | 0.01 ± 0.24 | 0.95 | -0.01 ± 0.03 | 0.64 |
| Diastolic blood pressure* | 0.04 ± 0.24 | 0.87 | -0.02 ± 0.03 | 0.45 |
| Fast glucose* | -0.31 ± 0.25 | 0.21 | -0.02 ± 0.03 | 0.60 |
| Urea* | -0.16 ± 0.25 | 0.52 | -0.04 ± 0.03 | 0.27 |
| Creatinine* | 1.85 ±0.22 | <.0001 | 0.03 ± 0.03 | 0.38 |
| Sodium* | -0.38 ± 0.23 | 0.10 | -0.06 ± 0.03 | 0.04 |
| Potassium* | 0.57 ± 0.26 | 0.030 | 0.04 ± 0.03 | 0.22 |
| Total bilirubin* | 0.17 ± 0.24 | 0.47 | -0.02 ± 0.03 | 0.59 |
| Conjugated bilirubin * | -0.19 ± 0.24 | 0.44 | -0.04 ± 0.03 | 0.27 |
| Total Cholesterol * | -0.04 ± 0.24 | 0.86 | -0.01 ± 0.03 | 0.71 |
| LDL cholesterol* | -0.07 ± 0.24 | 0.77 | -0.0002 ± 0.03 | 0.99 |
| Triglycerides* | 0.009 ± 0.24 | 0.97 | 0.01 ± 0.03 | 0.63 |
| Alkaline Phosphatase* | -0.12 ± 0.24 | 0.62 | 0.01 ± 0.03 | 0.69 |
| ASAT* | 0.76 ± 0.24 | 0.001 | -0.01 ± 0.03 | 0.72 |
| ALAT* | 0.50 ± 0.24 | 0.034 | -0.04 ± 0.03 | 0.18 |
| Gamma GT * | -0.21 ± 0.24 | 0.39 | -0.02 ± 0.04 | 0.63 |
| Erythrocytes* | 0.14 ± 0.22 | 0.52 | 0.006 ± 0.03 | 0.85 |
| Hematocrit* | 0.55 ± 0.27 | 0.039 | 0.03 ±0.04 | 0.45 |
| Hemoglobin * | 0.45 ±0.23 | 0.06 | 0.02 ± 0.03 | 0.43 |
| MCV * | 0.10 ± 0.05 | 0.038 | 0.005 ± 0.006 | 0.42 |
| MCH * | 0.47 ± 0.24 | 0.05 | 0.03 ± 0.03 | 0.39 |
| MCHC * | -0.07 ± 0.24 | 0.77 | 0.005 ± 0.03 | 0.88 |
| Leukocyte* | -0.20 ± 0.23 | 0.39 | 0.05 ± 0.03 | 0.09 |
| Platelet* | -0.23 ± 0.24 | 0.34 | -0.03 ± 0.03 | 0.29 |
| Neutrophil * | -0.30 ± 0.24 | 0.21 | 0.01 ± 0.03 | 0.70 |
| Lymphocyte* | 0.09 ± 0.24 | 0.70 | -0.009 ± 0.03 | 0.78 |
| Monocyte* | 0.75 ± 0.24 | 0.002 | 0.02 ± 0.03 | 0.45 |
| Eosinophil* | 0.11 ± 0.24 | 0.64 | -0.05 ± 0.03 | 0.11 |

The univariate analysis was performed on the global cohort. * corresponds to a factor that causes a coefficient increase of one standard deviation in the bivariate mixed models. Patient behavioural readouts were ALSFRS-r: Revised ALS functional rating scale, BMI: Body mass index, MMT: muscle strength measured by manual muscle testing and SVC: Slow Vital Capacity as a percent of the predicted value. Biofluid markers were CPK: creatine phosphokinase, LDL: Low Density Lipoprotein, ASAT: aspartate aminotransferase, ALAT: alanine aminotransferase, gamma-GT: gamma-glutamyltranspeptidase, MCV: mean corpuscular volume, MCH: mean corpuscular hemoglobin, MCHC: mean corpuscular hemoglobin concentration.

**Table e-3: Final model of baseline factors associated with ALSFRS-r at a given time and with progression**

|  | Effect on ALSFRS-r at fixed time or baseline | | Effect on ALSFRS-r progression | |
| --- | --- | --- | --- | --- |
| Factors at baseline | Coefficient  β ±SE | p | Coefficient  β ±SE | p |
| Time effect (month) | 0.21 (2.16) | 0.92 |  |  |
| Time since the onset of signs (month) | -0.05 (0.02) | 0.024* | 0.03 (0.004) | <.001** |
| El Escorial category (Definite vs Probable ALS) | -1.44 (0.43) | <0.001 |  |  |
| MMT^a^ | 2.80 (0.19) | <.001* | 0.17 (0.03) | <.001** |
| BMI^a^ | -0.52 (0.17) | 0.003* | 0.06 (0.03) | 0.038** |
| SVC^a^ | 0.78 (0.19 | <.001* | 0.08 (0.03) | 0.006** |
| Life quality^a^ | 0.76 (0.17) | <.001 |  |  |
| CPK^a^ | 0.55 (0.18) | 0.003 | - | - |
| MCV^a^ | 0.49 (0.18) | 0.005 | - | - |
| Sodium | -0.29 (0.17) | 0.10* | -0.05 (0.03) | 0.088** |

The multivariate analysis was performed on the global cohort using the final mixed model. β**^a^** indicates a factor that causes a coefficient increase of one standard deviation in the mixed model after backward selection. If an interaction was included in the multivariate model, the corresponding characteristic was also introduced, irrespective of its p-value. The allocated treatment group was included in the multivariate analysis as a forced variable to take into account the randomized design of the study. A backward selection (at a level of 0.05 for factors alone and 0.10 for their interactions with time) was performed manually to simplify the multivariable model by eliminating the irrelevant characteristics and interactions. *: factors that effect ALSFRS-r score at baseline. **: a significant interaction with time and the variable; a positive coefficient in the four factors indicate a lower decrease in ALSFRS-r. Sodium was associated with a worsening of ALSFRS-r (p<0.10). Analysis was adjusted for treatment effect.
